# Mental Health and Resilience Among the 85+ Lifestyle Leaders Throughout the COVID-19 Pandemic

**DOI:** 10.1093/geroni/igab046.1070

**Published:** 2021-12-17

**Authors:** Julie Miller

**Affiliations:** MIT, Cambridge, Massachusetts, United States

## Abstract

Recent research suggests older adults may be uniquely able to cope and cultivate psychological resilience during the COVID-19 pandemic. This presentation will describe how the Lifestyle Leaders’ overall mental health (including worries and experiences of social isolation and loneliness) and thoughts about the future have changed throughout the pandemic, as well as the ways in which they remained resilient throughout. For example, a survey in March 2020 indicated that 68% of Lifestyle Leaders were very or extremely worried about COVID-19, compared to only 33% in November 2020. Interviews with Lifestyle Leaders revealed that the pandemic led many to engage in more focused thinking about their own mortality and, for some, presented or compounded challenges of older age (e.g., widowhood, downsizing, etc.). The presentation will also highlight ways in which Lifestyle Leaders’ past experiences and current activities have contributed to their mental health and fortitude during the ongoing pandemic.

